# Iranian Journal of Basic Medical Sciences in times of the COVID-19 pandemic

**DOI:** 10.22038/IJBMS.2020.53529.12042

**Published:** 2021-01

**Authors:** Leila Arabi, Bizhan Malaekeh-Nikouei, Ali Roohbakhsh, BiBi Sedigheh Fazly Bazzaz

**Affiliations:** 1 Nanotechnology Research Center, Pharmaceutical Technology Institute, Mashhad University of Medical Sciences, Mashhad, Iran (Assistant Editor); 2 Pharmaceutical Research Center, Pharmaceutical Technology Institute, Mashhad University of Medical Sciences, Mashhad, Iran (Assistant Editor); 3 Biotechnology Research Center, Pharmaceutical Technology Institute, Mashhad University of Medical Sciences, Mashhad, Iran (Editor–in–Chief)

Since the beginning of 2020, the COVID-19 pandemic has affected people's lives throughout the world. After Severe Acute Respiratory Syndrome (SARS) in 2003 (1) and Middle East respiratory syndrome coronavirus (MERS-CoV) in 2012 (2), COVID-19 is considered as the third deadly pandemic due to a coronavirus family. Implementing a lockdown in many countries led to negative impacts on people's lives, businesses, and also the academic society (3). In times of this global stress, continuing research and publishing is considered very challenging. Despite the difficult and uncertain situation, we had an increased number of submitted manuscripts to the Iranian Journal of Basic Medical Sciences (IJBMS) compared to the last year (4), which implies the respectable level of its influence in the medical scientific community. 

We are excited to announce that Thomson Reuters (ISI) issued** 2.146 **as the impact factor of IJBMS for 2019.

This secured the journal’s position as one of the internationally prestigious scientific journals of Mashhad University of Medical Sciences (MUMS) (5). The upward trend of the impact factor indicates the quality of the multidisciplinary journal in the medical field (6).

**Figure 1:**
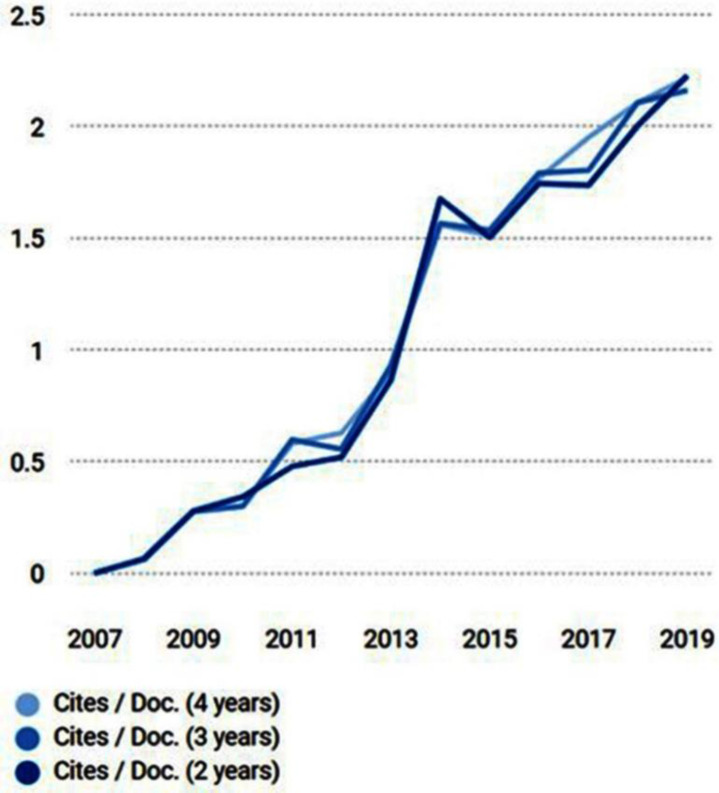
Citations per document (adopted from Scimago Journal & Country Rank)

Figure 1 displays the progress of the average number of times that published manuscripts in the past two, three, and four years have been cited in the current year. The two year line (dark blue) corresponds to the journal impact factor based on the Thomson Reuters metric.

Although the unprecedented COVID-19 pandemic circumstances led to some delays in the peer review process, no significant disruption in our regular editorial activities was encountered. We certainly tried to maintain our concerns and priority regarding the quality of research projects. Therefore, firstly we evaluated the submitted manuscripts for fitting the scope of the journal, the author guidelines, and the ethics and policies of the Committee on Publication Ethics (COPE). It was followed by focusing on the methodologic and scientific soundness of all accepted papers to avoid the so-called "wasted research", which was discussed in our last year's editorial (4). Based on that, we are convinced that the COVID-19 pandemic has not had a major negative impact on our services and publication in 2020. 

**Figure 2:**
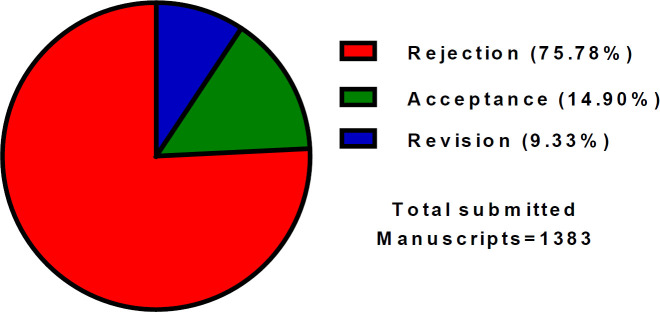
Overview of the percentages of submitted, rejected, and accepted manuscripts in 2020

The percentages of the number of submitted, rejected, and accepted manuscripts in 2020 are demonstrated in Figure 2.

It should be noted that it was not possible without the great contribution of national and international reviewers, who play a crucial role in this process. The reviewers’ thoughtful comments and recommendations help the further development of the manuscripts and assist the editorial team to reach the final decision in the best possible time. 

We would like to take this opportunity to express our sincere appreciation for our reviewers' assistance and dedicating time out of their busy schedules through the COVID-19 pandemic to improve the quality and credibility of our journal. We wish our readers and colleagues a healthy and safe NEW YEAR. With encouraging prospects for COVID-19 vaccines (7), hopefully the COVID-19 pandemic is defeated all over the world before long. 
